# Effect of Different Salinities on the Biochemical Properties and Meat Quality of Adult Freshwater Drum (*Aplodinotus grunniens*) During Temporary Rearing

**DOI:** 10.3390/antiox13101273

**Published:** 2024-10-21

**Authors:** Wanwen Chen, Sharifa Mohamed Miraji, Yu Tian, Xueyan Ma, Wu Jin, Haibo Wen, Gangchun Xu, Pao Xu, Hao Cheng

**Affiliations:** 1 Key Laboratory of Integrated Rice-Fish Farming Ecology, Ministry of Agriculture and Rural Affairs, Freshwater Fisheries Research Center, Chinese Academy of Fishery Sciences, Wuxi 214081, China; chenwanwen@ffrc.cn (W.C.); maxy@ffrc.cn (X.M.); jinw@ffrc.cn (W.J.); xugangchun@ffrc.cn (G.X.); xupao@ffrc.cn (P.X.); 2 Wuxi Fisheries College, Nanjing Agricultural University, Wuxi 214081, China; sherymiraj@gmail.com (S.M.M.); 13526711422@163.com (Y.T.); 3 Sino-US Cooperative International Laboratory for Germplasm Conservation and Utilization of Freshwater Mollusks, Freshwater Fisheries Research Center, Chinese Academy of Fishery Sciences, Wuxi 214081, China; 4 Department of Fisheries Development and Marine Resources, Ministry of Blue Economy and Fisheries, Zanzibar P.O. Box 774, Tanzania; 5 State Key Laboratory of Food Science and Resources, Jiangnan University, Wuxi 214122, China; haocheng@jiangnan.edu.cn; 6 School of Food Science and Technology, Jiangnan University, Wuxi 214122, China

**Keywords:** freshwater drum, salinity, antioxidants, meat quality

## Abstract

Salinity is a significant environmental component that affects the physiological state of aquatic species. This study aimed to investigate whether water salinity had an impact on the biochemical properties and meat quality of adult *Aplodinotus grunniens* during temporary rearing of 7 days. Salinity caused increased osmotic pressure and antioxidant enzyme activities of *Aplodinotus grunniens*, which were attributed to the increase in the content of alanine and glutamate. It raised the hardness and shear force with an increase in salinity, leading to an increase in water-holding capacity. Salinity enhanced the DHA ratio with a decrease in the atherosclerotic index and thrombosis index. Combined with the increase in flavor amino acids and nucleotides, salinity enhanced the umami taste of *Aplodinotus grunniens*. These findings suggest that temporary rearing in salinity might be a practical approach to improving the meat quality of adult *Aplodinotus grunniens*.

## 1. Introduction

Aquaculture is one of the fastest-emerging food-producing sectors globally and is increasingly flourishing [1]. Freshwater drum (*Aplodinotus grunniens*) is the only species in the genus of *Aplodinotus* that exclusively inhabits freshwater throughout its entire life and is widely distributed in North and Central America [2]. It has attracted great attention from both fishermen and consumers due to its fast growth rate, low oxygen resistance and strong adaptability, as well as its thick back muscles with non-intermuscular fish bones, high meat quality, and abundant nutrients [3]. In 2019, the Chinese Academy of Fishery Sciences’ Freshwater Fisheries Research Centre accomplished a significant milestone in the artificial breeding and cultivation of *Aplodinotus grunniens*. However, previous studies mainly pay attention to embryonic development [4], genetic diversity [5], fishing [6], and the effects of stress conditions such as high fat [7], hunger [8], and low temperature [9] on oxidative stress, lipid metabolism, intestinal microbial balance, and immune function of *Aplodinotus grunniens*. Little research has focused on the meat quality of *Aplodinotus grunniens*.

The meat quality of muscle is significantly affected by the nutritional composition and water-holding capacity, which might be associated with the physicochemical properties [10]. The cultural conditions are of great importance for the improvement of fish growing status and meat quality during aquaculture [11]. Salinity is a significant environmental component that has a direct impact on the physiological state of aquatic species [12]. Under salinity stress, energy metabolism rises to keep the osmotic balance in organisms, leading to changes in oxidation levels [13,14]. The growth and digestive enzyme activity were shown to be improved in juveniles of the fat snook (*Centropomus parallelus*) as the salinity increased [15]. In addition, salinity also has a great effect on the nutrients, texture, and taste of aquatic animals. For example, salinity influenced hardness, chewiness, gumminess, and the contents of flavor compounds in marine euryhaline crabs (*Scylla paramamosain*) [16]. Salinity also showed a significant effect on the content of total monounsaturated fatty acid and total n-3 polyunsaturated fatty acid in the gonad and liver tissues of rainbow trout [17]. It was found that the *Scylla paramamosain* had more unsaturated fatty acids in the high salinity group of 25‰ than the low salinity groups of 4‰ and 12‰. Additionally, the impacts of salinity on EPA and DHA are species specific [18]. Although salinity has been shown to affect the growth and quality of juvenile aquatic animals, the effect of short-term salinity treatment on the biochemical properties and meat quality of adult fish remains unclear [19].

In the present study, the effect of salinity on the biochemical properties and meat quality of *Aplodinotus grunniens* during temporary rearing was investigated. Osmotic pressure and antioxidant indicators were measured to explore the changes in blood and redox homeostasis in muscle under different salinity stress. All the biochemical properties were associated with the changes in free amino acids, fatty acids, nucleotides, texture, as well as water-holding capacity. This study may provide important guidelines and an efficient approach for the improvement of the meat quality of adult *Aplodinotus grunniens* through short-term salinity treatment during rearing.

## 2. Materials and Methods

### 2.1. Chemicals

Sodium chloride, copper (II) sulfate pentahydrate, potassium sulfate, potassium hydroxide sodium hydroxide, concentrated sulfuric acid, hydrochloric acid, boric acid, perchloric acid, trichloroacetic acid, methanol, boron trifluoride, n-hexane, and heparin were of analytical grade and purchased from Sinopharm Chemical Reagent Co., Ltd. (Shanghai, China). The malondialdehyde (MDA), catalase (CAT), glutathione peroxidase (GSH-Px), and total antioxidant capacity (T-AOC) detecting kits were purchased from Nanjing Jiancheng Bioengineering Company (Nanjing, China).

### 2.2. Experimental Fish and Rearing Conditions

The freshwater drums (*Aplodinotus grunniens*) with a body weight of 1.5 ± 0.25 kg were provided by the Chinese Academy of Fishery Sciences’ Freshwater Fisheries Research Centre. First, salinities of 4‰ (4 ppt), 8‰ (8 ppt), and 12‰ (12 ppt) were obtained by adding sodium chloride (NaCl) to the freshwater and confirmed by a SaltTestr 11 salinometer (Thermo Eutech Co., Ltd., Waltham, MA, USA). Freshwater was treated as the control (0 ppt). Then, a total of 48 *Aplodinotus grunniens* were randomly assigned to four groups with each group containing 12 fishes and cultured at Jiangyin City Shengang Co., Ltd. (Jiangyin, China) under the salinity of 0 ppt, 4 ppt, 8 ppt, and 12 ppt for seven days. Water temperature, pH, ammonia nitrogen, and nitrite were measured using a Palintest 7500 multi-parameter water quality analyzer (Palintest Co., Ltd., Gateshead, UK). The dissolved oxygen was determined by a HQ1130 portable water quality analyzer (HACH Co., Ltd., Loveland, CO, USA). Throughout the experiment, all the water quality parameters were determined every two days and kept as follows: water temperature of 24–26 °C, pH of 7.0–8.0, dissolved oxygen > 5 mg/L, ammonia nitrogen < 0.05 mg/L, and nitrite < 0.05 mg/L.

### 2.3. Sample Collection

After seven days of aquaculture, 12 *Aplodinotus grunniens* in each salinity group were collected. Blood samples of each group (*n* = 12) were collected from the caudal vein using a syringe and put into anticoagulation tubes of 10 mL. The white muscles above the lateral line and below the dorsal fin were collected. For enzyme activity analysis, the muscle samples of each group (*n* = 9) were frozen immediately in liquid nitrogen and stored at −80 °C before detection. The cooking loss, drip loss, freezing loss, and centrifugal loss were detected immediately after muscle collection (*n* = 6). The muscle samples of each group (*n* = 6) for the texture profile analysis were stored at 4 °C and detected within 24 h. As for the nutrition determination, which includes protein, lipid, amino acid, fatty acid, and nucleotide variables, the muscle samples of each group (*n* = 3) were stored at −80 °C before the analysis.

### 2.4. Biochemical Properties Analysis

#### 2.4.1. Morphological Index Analysis

The body weight, liver weight, viscera weight, and body length of each *Aplodinotus grunniens* (*n* = 12) were recorded. The condition factor (CF), hepatosomatic index (HSI), and viscerosomatic index (VSI) were calculated using the following formulas:

(1) $$\mathrm{Hepatosomatic}\;\mathrm{index}\;\left(\mathrm{HSI}\right)\left(\%\right)=\frac{\mathrm{Liver}\;\mathrm{weight}\;\left(g\right)}{\mathrm{Body}\;\mathrm{weight}\;\left(g\right)}\times 100$$

(2) $$\mathrm{Viscerosomatic}\;\mathrm{index}\;\left(\mathrm{VSI}\right)\left(\%\right)=\frac{\mathrm{Viscera}\;\mathrm{weight}\;\left(g\right)}{\mathrm{Body}\;\mathrm{weight}\;\left(g\right)}\times 100$$

(3) $$\mathrm{Condition}\;\mathrm{factor},\;\mathrm{CF},\;g/{\mathrm{cm}}^{3}=\frac{\mathrm{Body}\;\mathrm{weight}\;\left(g\right)}{{\mathrm{Body}\;\mathrm{length}}^{3}\left({\mathrm{cm}}^{3}\right)}\times 100$$

#### 2.4.2. Osmotic Pressure Analysis

Blood samples were centrifuged at 5000 rpm at 4 °C for 10 min to extract the plasma after collection immediately. Then, the osmotic pressure of plasma was detected using an Osmomat 030 cryoscopic osmometer (Gonotec GmbH, Berlin, Germany).

#### 2.4.3. Oxidative Stress Analysis

First, 0.1 g of muscle samples was mixed with 0.9 mL of normal saline using a Scientz-48L high throughput tissue grinder (Xinzhi Biotechnology Co., Ltd., Ningbo, China) and centrifuged at 3000 rpm at 4 °C for 10 min. The supernatant was used for MDA, CAT, GSH-Px, and T-AOC analysis according to the protocol using detection kits. MDA was measured using a thiobarbituric acid (TBA) method. The ammonium molybdate-chromogenic method was applied to determine CAT. GSH-Px assay was performed based on a colorimetric method. T-AOC was detected by the ABTS method.

### 2.5. Water-Holding Capacity and Texture Property Analysis

The water-holding capacity was determined by centrifugal loss, drip loss, thaw loss, and cooking loss according to the method described by Li et al. [20] with minor modifications. The texture profile analysis (TPA) and shear force analysis were also conducted by the methods described previously by Song et al. [21].

#### 2.5.1. Determination of Centrifugal Loss

Briefly, 5 g of the muscle squares was wrapped in filter paper and accurately weighed as W_1_. Then, the muscle samples were centrifuged at 4000 rpm under 4 °C for 30 min. Then, the surface moisture of the samples was absorbed by the filter paper again and the samples were weighed as W_2_. The centrifugal loss was calculated as follows:

(4) $$\mathrm{Centrifugal}\;\mathrm{loss}\;\left(\%\right)=\frac{W_{1}-W_{2}}{W_{1}}\times 100$$

#### 2.5.2. Determination of Drip Loss

The surface moisture of the muscle squares (5 g) was absorbed by filter paper and weighted (W_3_). Then, the samples were put in self-sealing polyethylene bags and stored in a refrigerator at 4 °C. After 24 h, they were taken out until they reached room temperature. The samples were wrapped in filter paper and reweighted as W_4_. The drip loss was identified using the following formula:

(5) $$\mathrm{Drip}\;\mathrm{loss}\;\left(\%\right)=\frac{W_{3}-W_{4}}{W_{3}}\times 100$$

#### 2.5.3. Determination of Cooking Loss

After wrapping the surface moisture, the weight of sample squares (5 g) was recorded as W_5_. The sample squares were put in boiling water. After 5 min, they were cooled down to room temperature, and the surface of the sample was wrapped in filter paper. Then, the samples were reweighted as W_6_. The calculation formula for cooking loss was as follows:

(6) $$\mathrm{Cooking}\;\mathrm{loss}\;\left(\%\right)=\frac{W_{5}-W_{6}}{W_{5}}\times 100$$

#### 2.5.4. Determination of Freezing Loss

The weight of muscle samples was determined as W_7_ and the samples were put in self-sealing polyethylene bags. The sample squares were then stored in a refrigerator at −20 °C. After 24 h, they were taken out and thawed to room temperature. The surface moisture of the samples was absorbed by filter paper and the weight of the samples was obtained (W_8_). The freezing loss was evaluated as follows:

(7) $$\mathrm{Freezing}\;\mathrm{loss}\;\left(\%\right)=\frac{W_{7}-W_{8}}{W_{7}}\times 100$$

#### 2.5.5. Texture Profile Analysis

Texture profile analysis (TPA) was determined according to the method described previously with some modifications [22]. The muscle samples were cut into 2 cm × 2 cm × 1.5 cm pieces and measured using a Texture Analysis XTPlus physical property analyzer equipped with a P/5 cylinder probe (Stable Micro Systems, Ltd., Surrey, UK). The test parameters were set as follows: pre-test speed of 2 mm/s, test speed of 1 mm/s, post-test speed of 2 mm/s, trigger force of 5 g, interval of 10 s, and test deformation of 60%. The parameters including hardness, springiness, cohesiveness, gumminess, chewiness, and resilience were recorded.

#### 2.5.6. Shear Force

The shear force of samples was determined using a Texture Analysis XTPlus physical property analyzer with an A/MORS probe (Stable Micro Systems, Surrey, UK). The muscle samples were cut into 2 cm × 3 cm × 1 cm pieces. The test conditions were set as follows: pre-test speed of 2 mm/s, test speed of 2 mm/s, post-test speed of 2 mm/s, trigger force of 5 g, and compress deformation of 50%.

### 2.6. Quantification of Nutrition and Taste Substances

#### 2.6.1. Chemical Composition Analysis

The chemical compositions including moisture, ash, crude protein, and crude fat contents of *Aplodinotus grunniens* muscle samples were examined according to the Association of Official Analytical Chemists (AOAC). The moisture content was examined by drying the samples at 105 °C until the weight reached a constant (AOAC Method 985.2912). The ash content was performed in a muffle furnace at 550 °C until a constant weight was obtained (AOAC Method 923.03). Crude protein content was determined by the Kjeldahl method (AOAC Method 925.09). Crude fat content was examined using the Soxhlet apparatus (AOAC Method 932.06).

#### 2.6.2. Free Amino Acid Analysis

Free amino acids were analyzed according to the method described by Wen et al. with some modifications [23]. First, 2 g of the muscle samples was mixed with 25 mL of 5% trichloroacetic acid (TCA) and treated using ultrasound for 30 min. The mixture was kept for 3 h and then filtered by double filter paper. The filtrates were then centrifugated at 10,000× *g* for 10 min and the supernatant was filtered through a 0.22 μm membrane. The free amino acid content was determined quantitatively by using an S433D automatic amino acid analyzer (Sykam, Eresing, Germany).

#### 2.6.3. Fatty Acid Analysis

Fatty acid compositions were analyzed according to the methods reported by Alahmad et al. with minor modifications [24]. The crude fat was extracted by using a hexane–methanol (2:1, *v*/*v*) solution for 12 h. Then, the crude fat was treated through methyl esterification. Briefly, 0.5 g of crude fat was mixed with 2 mL 0.5 M sodium hydroxide–methanol solution and kept in a water bath at 60 °C for 30 min. After cooling, 2 mL boron trifluoride diethyl ether–methanol solution was added to the mixture and continued to react at 60 °C for 30 min. Thereafter, 2 mL n-hexane was added to the cooled mixture. The upper organic phase was filtered through a 0.22 μm membrane and then determined by using GC-2030AF gas chromatography (Shimadzu, Kyoto, Japan). The atherosclerotic index (AI) and thrombosis index (TI) were also calculated using the following formulas as previously reported [25]:

(8) $$S_{\mathrm{AI}}=\frac{4\times S_{C_{14:0}}+S_{C_{16:0}}}{\sum S_{\mathrm{MUFA}}+\sum_{n-6}S_{\mathrm{PUFA}}+\sum_{n-3}S_{\mathrm{PUFA}}}$$

(9) $$S_{\mathrm{TI}}=\frac{S_{C_{14:0}}+S_{C_{16:0}}+S_{C_{18:0}}}{0.5\times \sum S_{\mathrm{MUFA}}+0.5\times \sum_{n-6}S_{\mathrm{PUFA}}+\frac{\sum_{n-3}S_{\mathrm{PUFA}}}{\sum_{n-6}S_{\mathrm{PUFA}}}+3\times \sum_{n-3}S_{\mathrm{PUFA}}}$$

#### 2.6.4. Nucleotide Determination

The nucleotide content of muscles was also evaluated according to the methods described by Du et al. with minor modifications [11]. Muscle samples (5 g) and 10 mL of 10% perchloric acid were homogenized at 8000 rpm for 1 min and then treated using ultrasound for 5 min. The extracts were centrifuged at 5000× *g* for 15 min at 4 °C and filtered with filter paper. Then, 5 mL of 5% perchloric acid was used to wash the precipitation twice and the filtrate was combined. The pH value of the supernatant was adjusted to 6.0 ~ 6.5 using KOH solution at a concentration of 6 mol/L. Finally, 1.5 mL of the supernatant was centrifuged at 10,000 rpm for 10 min at 4 °C. The supernatant was further filtered through a 0.22 μm membrane and analyzed by a Waters e2695 high-performance liquid chromatography (Waters Co., Ltd., Milford, MA, USA). The contents of adenosine-5′-monophosphate (AMP), guanosine-5′-monophosphate (GMP), and inosine-5′-monophosphate (IMP) were identified and quantified based on the retention times and peak areas of the standards. The taste activity value (TAV) was calculated as the ratio between the absolute concentration value of taste compounds and their threshold values. The equivalent umami concentration (EUC) also was calculated according to the formula reported by Zhang et al. as follows [26].

(10) $$Y=\sum a_{i}b_{i}+1218(\sum a_{i}b_{i})(\sum a_{j}b_{j})$$

where Y is the EUC expressed in MSG equivalent concentration (g/100 g). a_i_ is the concentration of each umami amino acid (Glu or Asp) (g/100 g). b_i_ is the relative umami concentration of each umami amino acid (Glu, 1; Asp, 0.077). 1218 is a synergistic constant. a_j_ is the concentration of each nucleotide (IMP, GMP, or AMP) (g/100 g). b_j_ is the relative umami concentration of each nucleotide (IMP, 1; GMP, 2.3; AMP, 0.18).

### 2.7. Statistical Analysis

The data were presented as mean value ± standard deviation (SD). Statistical comparisons for more than two groups were performed by one-way ANOVA followed by Duncan’s test using the SPSS software 20.0 (SPSS Institute, New York, NY, USA). Differences were considered statistically significant at *p* < 0.05.

## 3. Results and Discussion

### 3.1. Biochemical Properties

#### 3.1.1. Morphological Index Analysis

The hepatosomatic index (HSI), viscerosomatic index (VSI), and condition factor (CF) of *Aplodinotus grunniens* are shown in Table 1. The HSI significantly decreased from 1.140% to 0.863% as the salinity increased from 0 ppt to 12 ppt. It has been reported that the glycolysis process can be affected by the culture environment due to the requirement of more energy to balance their body fluid, leading to the release of lipids and glucose from the liver cells [27]. The changes in HSI might be related to the decrease in liver weight induced by exposure to a salinity environment. However, VSI also exhibited a decrease in salinity groups (4, 8, 12 ppt) as compared to the control group (0 ppt), while no significant difference occurred among different salinity groups. CF is an indication of the well-being of an organism. The CF at the high salinity of 12 ppt reached 2.279 g/cm^3^, which was higher than the control group (0 ppt) and low salinity groups (4 and 8 ppt). A previous study revealed that the CF is relatively higher for the fin fish species collected from the Hugli estuary (relatively hypo saline) compared to those collected from the Matla estuarine water [28]. Moreover, the study of blue tilapia *Oreochromis aureus* also showed that the CF was increased significantly in the high salinity group as compared to the low salinity group [29]. Our results were consistent with the previous research.

#### 3.1.2. Osmotic Pressure Analysis

**Figure 1:**
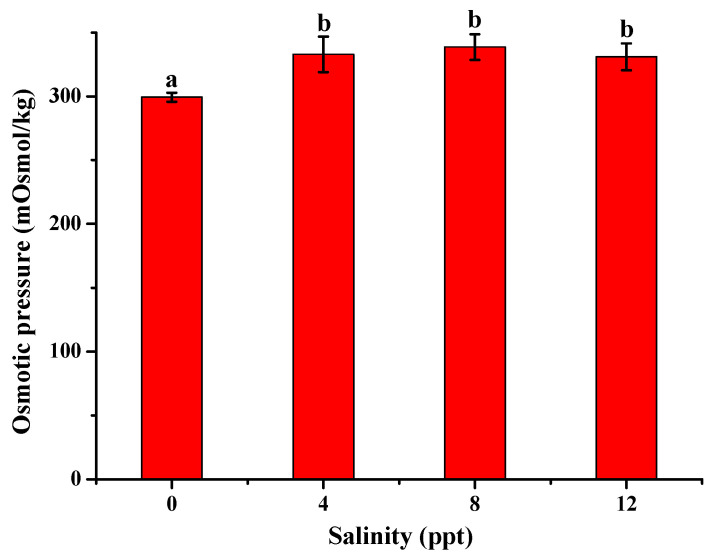
Effect of different salinity levels on the osmotic pressure of *Aplodinotus grunniens* for 7 days (*n* = 12). Different letters represent significant differences between different salinity groups at *p* < 0.05.

Osmotic pressure is determined by the total concentration of solutes in the body fluid, which is affected by the inorganic ions, especially the inorganic ions of Na^+^ and Cl^−^ [30]. As shown in Figure 1, the osmotic pressure of *Aplodinotus grunniens* increased from 299 mOsmol/kg to 333 mOsmol/kg as the salinity increased from 0 to 4 ppt. The increase in the osmotic pressure might be related to the increase of inorganic ionic concentration in the serum of *Aplodinotus grunniens*. No significant difference was observed between the salinity groups (4, 8, 12 ppt), indicating that *Aplodinotus grunniens* can maintain their osmotic pressure at the salinity range from 4 to 12 ppt.

The changes in plasma osmolality presented in our study were in line with the results reported by Huong et al., that the osmotic pressure in striped catfish fingerling size increased as salinity increased [31]. Increasing osmotic pressure was also found in striped catfish due to salinity [32].

#### 3.1.3. Oxidative Stress Analysis

**Figure 2:**
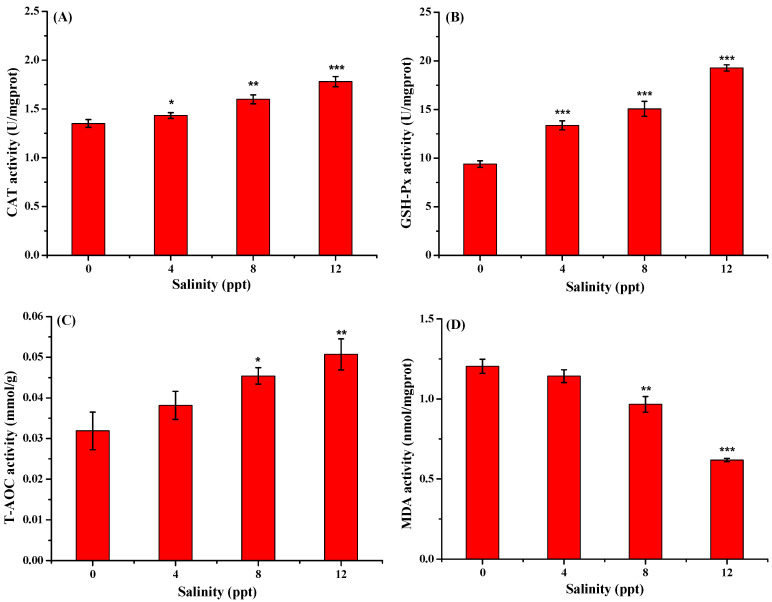
Effect of salinity on the CAT (**A**), GSH-Px (**B**), T-AOC (**C**), and MDA (**D**) activities in *Aplodinotus grunniens* muscle (*n* = 9). Values marked with *: *p* < 0.05, **: *p* < 0.01, and ***: *p* < 0.001 indicated significant differences when compared to the control group.

The antioxidant enzyme activities of *Aplodinotus grunniens* muscle at different salinity levels are illustrated in Figure 2. The CAT activity significantly increased from 1.352 to 1.433, 1.599, and 1.781 U/mgprot as the salinity increased from 0 to 4, 8, 12 ppt, respectively. The GSH-Px activity showed a similar trend to the CAT activity, which ranged from 9.387 mol/mgprot in the control group to 19.277 nmol/mgprot in the salinity group of 12 ppt. As for T-AOC activity, a significant increase can also be observed in the salinity groups of 8 and 12 ppt as compared to the control group. The results indicated that both the CAT, GSH-Px, and T-AOC activities increased significantly with salinity increasing. According to the previous study, an increase in enzyme activity is a response to oxidative stress [33].

As shown in Figure 2D, the MDA activity reached 1.204, 1.143, 0.967, and 0.618 nmol/mgprot at the salinity of 0, 4, 8, and 12 ppt, respectively. It can be seen that the MDA activity decreased significantly as the salinity increased. The same results were observed in the rainbow trout (*Oncorhynchus mykiss*) with the highest MDA content in the control group and the lowest MDA content in the salinity groups, suggesting that rainbow trouts were healthier in the salinity group compared to those in the control groups [34]. Wang et al. reported that low salinity played a protective role toward cadmium exposure in juvenile *Takifugu obscurus* by reducing the reactive oxygen species and MDA levels toward cadmium exposure [35], while their further study found that MDA content significantly increased first and then decreased in newly hatched *Takifugu obscurus* larvae after exposure to the high salinity of 10 and 20 ppt [36]. Based on the above results, the increase in antioxidant enzyme activities in *Aplodinotus grunniens* muscle after exposure to salinity might be attributed to the activation of the antioxidant system to reduce oxidative stress by eliminating peroxide products such as MDA and absorbing free radicals.

### 3.2. Water-Holding Capacity and Texture Property Analysis

#### 3.2.1. Water-Holding Capacity Analysis

**Figure 3:**
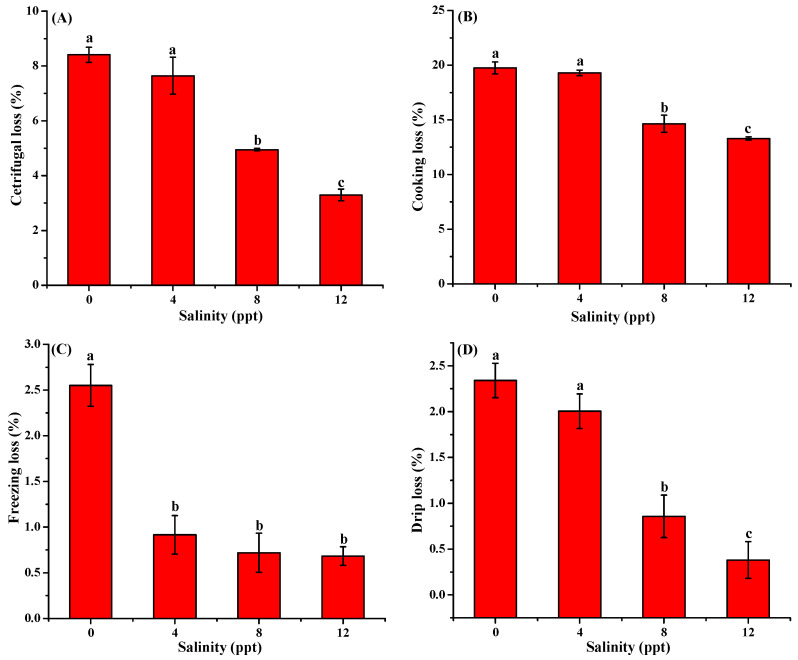
Effect of salinity on the centrifugal loss (**A**), cooking loss (**B**), freezing loss (**C**), and drip loss (**D**) of *Aplodinotus grunniens* muscle (*n* = 6). Different letters represent significant differences between different salinity groups at *p* < 0.05.

The effect of salinity on the water-holding capacity of *Aplodinotus grunniens* muscle was displayed in Figure 3. The results showed that the centrifugal loss, cooking loss, freezing loss, and drip loss were significantly decreased in the salinity groups as compared to the control group, indicating the increase in water-holding capacity. Additionally, except for the freezing loss, the centrifugal loss, cooking loss, and drip loss also decreased as the salinity increased from 4 to 12 ppt. The results were similar to the changes in water-holding capacity in largemouth bass under high water salinity [11].

The water-holding capacity refers to the physical binding force of the *Aplodinotus grunniens* muscle to water under external force, which is related to the taste and quality of fish muscle [37]. According to the previous study, a high rate of muscle water loss is associated with the loss of soluble protein, resulting in rough and poor mouth feeling in meat [29]. In the present study, higher water-holding capacity induced by salinity might be attributed to the reaction between the Cl^-^ ion and proteins to increase their negative electrical charges and cause proteins to undergo swelling. The increase in the capacity to hold water would also preserve the quality of meat throughout storage and cooking.

#### 3.2.2. Texture Profile Analysis

The texture properties of hardness, springiness, cohesiveness, gumminess, chewiness and resilience are shown in Table 1. A significant increase was observed in hardness as the salinity increased. It reached 624.601, 692.884, and 715.955 N at the salinity level of 4, 8, and 12 ppt, which was higher than that in the control group (535.128 N). The gumminess and chewiness also increased when the salinity increased from 0 to 8 ppt, and then showed a decrease in the salinity of 12 ppt. However, no significant difference occurred between different groups. A previous study reported that the hardness, chewiness, gumminess, resilience, and springiness of grass carp (*Ctenopharyngodon idellus*) were higher than those in the control group [38]. The increased hardness in our study might be associated with the higher water-holding capacity induced by the salinity.

#### 3.2.3. Shear Force Analysis

The effect of salinity on the shear force of *Aplodinotus grunniens* muscle is also presented in Table 1. The shear force significantly increased from 2.670 to 3.420 N as the salinity increased from 0 to 12 ppt, while no significant difference was observed at the salinity of 4 and 8 ppt as compared to the control group. The results were similar to the previous report in which a higher shear force was observed in the salinity groups in comparison to the control group. The increase in shear force represents the decrease in tenderness, which was correlated with juiciness [11]. It was also revealed that the higher shear force is positively related to the harder meat [39]. Thus, the results obtained in the shear force analysis were consistent with the data in the texture profile analysis.

### 3.3. Quantification of Nutrition and Taste Substances

#### 3.3.1. Chemical Composition Analysis

The chemical composition values including moisture, ash, crude protein, and crude lipid content are displayed in Table 1. The results revealed that salinity did not cause any significant differences in moisture, ash, crude protein, and crude lipid content. This was similar to the result in a previous report that salinity did not change the proximate composition of mud crab muscle and Pacific white shrimp muscle [16,40]. Additionally, no significant difference was noted in the composition of grass carp muscle cultured at different salinities [41].

#### 3.3.2. Free Amino Acid Analysis

In this study, 17 free amino acids of the *Aplodinotus grunniens* muscle at different salinity levels were analyzed, including threonine (Thr), valine (Val), methionine (Met), phenol (Phe), isoleucine (Ile), leucine (Leu), lysine (Lys), aspartate (Asp), glutamate (Glu), glycine (Gly), alanine (Ala), serine (Ser), histidine (His), arginine (Arg), cystine (Cys), proline (Pro), and tyrosine (Tyr). As shown in Table 2, the content of Thr, Ala, Glu, Ser, Pro, and His in the salinity groups showed significant differences as compared to the control groups. As the salinity increased from 0 to 4, 8, and 12 ppt, the content of total amino acid (TAA) significantly increased from 0.208 to 0.312, 0.329, and 0.376 g/100 g with the essential amino acids (EAAs) increasing from 0.042 to 0.056, 0.060, and 0.060 g/100 g. Additionally, the content of flavor amino acids (FAA)s also increased with the increase in salinity, which was around 1.93 times higher in salinity of 12 ppt than that in the control group. The results suggested that salinity can enhance the flavor of *Aplodinotus grunniens* muscle. Similar results were obtained in the changes in amino acids in Blue Tilapia (*Oreochromis aureus*) raised under salinity conditions [29]. According to a previous study, the increased FAA content can also be positively associated with the rise in Na^+^ and Cl^-^ [42]. However, another study revealed that low salinity weakened the umami taste and aroma characteristics of crab flesh associated with decreased contents of free amino acids in flesh [16].

Salinity is a significant environmental component that can raise the total free amino acid content and maintain the equilibrium of osmoregulation in aquatic animals [42]. It has been reported that exposure to seawater caused notable changes in the metabolism of amino acids in *Salvelinus alpinus* as compared to freshwater. Associated with the production of umami, bitter, and sweet flavor-related amino acids was a rise in the activity of the amino acid related to the metabolism of enzymes [19]. Moreover, the amino acids that increased due to salinity also function as energy substrates, which are essential for the osmoregulation energy supply. For example, Glu is a major organic osmolyte in fish. It is a member of the “Glu family” that can be transaminated into Glu and Glu transamination, contributing to the oxidation pathways in the tissues of fishes. Ala is a superior substrate for gluconeogenesis and is considered the principal energy provider when fish are exposed to long-term oxidative stress [43]. In the present study, the increase in the content of Ala and Glu is also consistent with the rise in antioxidant enzyme activities, as well as the results in osmotic pressure analysis.

#### 3.3.3. Fatty Acid Analysis

The fatty acid compositions in *Aplodinotus grunniens* muscle at different concentrations of salinity are displayed in Table 3. The ratio of eicosanoic acid (C20:0) was higher in the salinity group at 4 ppt as compared to the control and other salinity groups, whereas the total saturated fatty acids (SFAs) showed a slight decrease as the salinity increased. However, no significant difference occurred in SFAs among the groups. The unsaturated fatty acids (UFAs), especially polyunsaturated fatty acids (PUFAs), also exhibited an increase in the salinity groups as compared to the control group. PUFAs, such as eicosadienoic acid (C20:2n-6) and docosahexaenoic acid (C22:6n-3, DHA), significantly increased (*p* < 0.05) in salinity groups than in the controlled group. DHA is essential for proper visual and neurological postnatal development [44]. The DHA ratio reached 5.666%, 5.325%, and 5.322% in the salinity groups of 4, 8, and 12 ppt, which was higher in comparison to the control group (5.015%). Moreover, the atherosclerotic index (AI) showed a decrease from 0.427 in the control group to 0.405, 0.401, and 0.401 in the salinity groups of 4, 8, and 12 ppt, with the same trends in the thrombosis index (TI), which decreased from 0.442 to 0.409, 0.416, and 0.416, respectively.

UFAs are one of the basic nutrients found in meat and are crucial to numerous biological processes in vertebrates. Previous studies have reported that PUFAs increased in Nile tilapia under salinity conditions as compared to freshwater [13]. Dong et al. also found that EPA and DHA were much more abundant in the salinity group than in the freshwater group in Japanese sea bass (*Lateolabrax Japonicus*) [45]. The AI and TI are nutritional indices that can be used to evaluate the potential effects of fatty acid compositions on cardiovascular health [25]. Thus, the increased UFA ratio and decreased AI and TI values in our study suggested that temporary rearing in a proper salinity environment is a practical approach to enhance PUFA deposition, which improves the nutritional value of *Aplodinotus grunniens* meat. In addition, the consumption of *Aplodinotus grunniens* meat reared under salinity conditions might reduce the risk of coronary heart disease.

#### 3.3.4. Nucleotide Determination

Table 4 presents the effect of salinity on the nucleotide content in the *Aplodinotus grunniens* muscle. No significant changes were observed in the content of AMP. It should be pointed out that both the IMP and GMP content increased significantly with an increase in salinity. As compared to the control group (1.674 mg/100 g), the content of GMP significantly increased to 1.986, 2.135, and 2.453 mg/100 g in the salinity groups of 4, 8, and 12 ppt. A similar trend was also observed in the content of IMP ranging from 41.352 to 164.468 mg/100 g. The result was also comparable with the findings in marine euryhaline crabs (*Scylla paramamosain*) and largemouth bass [11,16]. The TAV of IMP reached 1.654, 2.295, 5.088, and 6.579 at the salinity of 0, 4, 8, and 12 ppt, which was far greater than 1, indicating that IMP was the dominant 5′-nucleotide in the *Aplodinotus grunniens* muscle. Additionally, the EUC calculated in the salinity group of 4, 8, and 12 ppt was 1.829, 3.850, and 6.534 g MSG/100 g, respectively, which was higher than the taste threshold of MSG (0.030 g/100 mL).

IMP, GMP, and AMP are flavor nucleotides that typically contribute to the sweetness and umami flavor of aquatic foods. It has been reported that IMP and GMP can contribute to a meaty flavor and also exhibit a stronger umami-enhancing effect than MSG [46]. Thus, the salinity might increase the meaty flavor and umami taste in the *Aplodinotus grunniens* muscle. Moreover, the interaction between FAA and nucleotides, as well as IMP and GMP, could exert umami synergistic effects, leading to an increase in the umami taste [47]. The results obtained from EUC values confirmed that the umami taste is very intense in the *Aplodinotus grunniens* muscle and can be improved by temporary rearing under salinity conditions.

## 4. Conclusions

In this study, the effect of different salinities on the biochemical properties of adult freshwater drum (*Aplodinotus grunniens*) during seven days of temporary rearing was investigated, which was also associated with the changes in the meat quality. Our results revealed that the salinity environment induced a decrease in the hepatosomatic index and an increase in the osmotic pressure in adult *Aplodinotus grunniens*. It caused an increase in the antioxidant enzyme activities of *Aplodinotus grunniens* muscle, including CAT, GSH-Px, and T-AOC activities, which was consistent with the increase in the content of Ala and Glu. Salinity also increased the meat quality of *Aplodinotus grunniens* through the improvement of water-holding capacity and texture. Additionally, salinity improved the nutritional value of *Aplodinotus grunniens* through increasing the UFA ratio and decreasing AI and TI values. Moreover, temporary rearing of *Aplodinotus grunniens* increased the content of FAAs, TAAs, IMP, and GMP, resulting in an enhanced umami taste as the salinity increased. The present study may be useful for guiding the improvement of the flesh quality of adult *Aplodinotus grunniens* through short-time rearing. However, further research is needed to explore the potential physiological and biochemical pathways affected by salinity, revealing the underlying mechanisms of salinity temporary rearing to improve the meat quality of adult *Aplodinotus grunniens*.

## Figures and Tables

**Table 1:** The effect of salinity on the morphological index, chemical compositions, texture properties, and shear force of *Aplodinotus grunniens*.

Index	Salinity (ppt)			
	0	4	8	12
Hepatosomatic index (%)	1.140 ± 0.080 ^a^	0.985 ± 0.109 ^b^	0.996 ± 0.104 ^bc^	0.863 ± 0.088 ^c^
Viscerosomatic index (%)	9.096 ± 0.438 ^a^	7.400 ± 0.467 ^b^	8.546 ± 0.837 ^a^	7.675 ± 1.262 ^a^
Condition factor (g/cm^3^)	1.345 ± 0.034 ^a^	1.341 ± 0.031 ^a^	1.544 ± 0.279 ^a^	2.279 ± 0.155 ^b^
Hardness (g)	535.128 ± 27.428 ^a^	624.601 ± 101.990 ^ab^	692.884 ± 16.394 ^b^	715.955 ± 90.001 ^b^
Springiness	0.731 ± 0.030	0.745 ± 0.019	0.750 ± 0.049	0.759 ± 0.049
Cohesiveness	0.453 ± 0.071	0.454 ± 0.033	0.471 ± 0.043	0.496 ± 0.036
Gumminess	238.641 ± 89.345	268.713 ± 85.751	322.551 ± 23.506	273.523 ± 8.724
Chewiness	193.141 ± 32.307	206.085 ± 49.714	229.440 ± 12.677	224.687 ± 42.349
Resilience	0.271 ± 0.086	0.238 ± 0.028	0.288 ± 0.013	0.292 ± 0.044
Shear force (N)	2.670 ± 0.069 ^a^	2.687 ± 0.445 ^a^	2.793 ± 0.381 ^ab^	3.420 ± 0.418 ^b^
Moisture (%)	71.367 ± 1.369	72.468 ± 1.038	71.066 ± 0.172	72.340 ± 0.471
Ash (%)	1.050 ± 0.059	1.057 ± 0.039	1.085 ± 0.072	1.117 ± 0.073
Crude protein (%)	18.805 ± 1.149	19.119 ± 1.140	19.303 ± 0.206	19.287 ± 0.554
Crude lipid (%)	29.297 ± 1.983	29.810 ± 1.026	31.273 ± 1.440	31.330 ± 2.008

Note: Data are presented as means ± SD (Hepatosomatic index, viscerosomatic index, condition factor, *n* = 12), (Hardness, springiness, cohesiveness, gumminess, chewiness, resilience, shearing force, *n* = 6), (Moisture, ash, crude protein, crude lipid, *n* = 3). Different letters represent significant differences between different salinity groups at *p* < 0.05.

**Table 2:** Effect of salinity on the free amino acid compositions in *Aplodinotus grunniens* muscle.

Free Amino Acid (g/100 g Wet Basis)	Salinity (ppt)			
	0	4	8	12
Leucine (Leu) *	0.004 ± 0.001	0.005 ± 0.001	0.006 ± 0.001	0.005 ± 0.001
Lysine (Lys) *	0.011 ± 0.004	0.018 ± 0.004	0.018 ± 0.004	0.019 ± 0.003
Valine (Val) *	0.006 ± 0.001	0.006 ± 0.002	0.007 ± 0.001	0.006 ± 0.001
Phenol (Phe) *	0.003 ± 0.001	0.002 ± 0.000	0.003 ± 0.000	0.002 ± 0.000
Isoleucine (Ile) *	0.003 ± 0.001	0.003 ± 0.000	0.004 ± 0.001	0.003 ± 0.000
Threonine (Thr) *^/^**	0.012 ± 0.002 ^a^	0.021 ± 0.002 ^b^	0.021 ± 0.005 ^b^	0.023 ± 0.004 ^b^
Methionine (Met) *	0.002 ± 0.001	0.002 ± 0.000	0.002 ± 0.000	0.002 ± 0.001
Alanine (Ala) **	0.029 ± 0.003 ^a^	0.056 ± 0.010 ^ab^	0.077 ± 0.039 ^b^	0.086 ± 0.021 ^b^
Glycine (Gly) **	0.084 ± 0.012	0.126 ± 0.053	0.117 ± 0.038	0.145 ± 0.046
Glutamate (Glu) **	0.017 ± 0.005 ^a^	0.022 ± 0.006 ^ab^	0.023 ± 0.007 ^ab^	0.030 ± 0.004 ^b^
Aspartate (Asp) **	0.011 ± 0.005	0.012 ± 0.003	0.011 ± 0.002	0.011 ± 0.002
Serine (Ser) **	0.003 ± 0.000 ^a^	0.006 ± 0.000 ^ab^	0.007 ± 0.004 ^ab^	0.008 ± 0.001 ^b^
Proline (Pro)	0.005 ± 0.002 ^a^	0.009 ± 0.003 ^ab^	0.009 ± 0.000 ^ab^	0.010 ± 0.002 ^b^
Histidine (His)	0.007 ± 0.002 ^a^	0.015 ± 0.002 ^b^	0.015 ± 0.008 ^b^	0.018 ± 0.005 ^b^
Cystine (Cys)	0.001 ± 0.000	0.000 ± 0.000	0.000 ± 0.000	0.001 ± 0.000
Arginine (Arg)	0.001 ± 0.000	0.002 ± 0.001	0.001 ± 0.000	0.001 ± 0.000
Tyrosine (Tyr)	0.009 ± 0.004	0.009 ± 0.003	0.009 ± 0.005	0.009 ± 0.005
Total amino acids (TAAs)	0.208 ± 0.025 ^a^	0.312 ± 0.061 ^ab^	0.329 ± 0.093 ^ab^	0.376 ± 0.060 ^b^
Essential amino acids (EAAs)	0.042 ± 0.006 ^a^	0.056 ± 0.008 ^ab^	0.060 ± 0.010 ^b^	0.059 ± 0.007 ^b^
Flavor amino acids (FAAs)	0.157 ± 0.024 ^a^	0.242 ± 0.057 ^ab^	0.256 ± 0.085 ^ab^	0.301 ± 0.059 ^b^

Note. Data are presented as means ± SD (*n* = 3). * represents the essential amino acids; ** indicates the flavor amino acids. Different letters represent significant differences between different salinity groups at *p* < 0.05.

**Table 3:** Effect of salinity on the fatty acid compositions in *Aplodinotus grunniens* muscle.

Fatty Acid (%)	Salinity (ppt)			
	0	4	8	12
C14:0	2.221 ± 0.058	2.115 ± 0.274	2.062 ± 0.120	2.012 ± 0.093
C15:0	0.220 ± 0.018	0.232 ± 0.036	0.215± 0.004	0.211 ± 0.009
C16:0	22.108± 0.738	21.261 ± 0.145	21.249 ± 1.437	21.439 ± 0.779
C17:0	0.213 ± 0.020	0.230 ± 0.036	0.202 ± 0.019	0.199 ± 0.011
C18:0	2.268 ± 0.044	2.435 ± 0.375	2.275 ± 0.217	2.299± 0.210
C20:0	0.129 ± 0.012 ^a^	0.147 ± 0.012 ^b^	0.135 ± 0.002 ^ab^	0.133 ± 0.002 ^ab^
C22:0	0.209 ± 0.036	0.224 ± 0.048	0.207 ± 0.032	0.213 ± 0.042
∑Saturated fatty acids (SFAs)	27.366 ± 0.653	26.644 ± 0.092	26.344 ± 1.550	26.506 ± 0.853
C14:1	0.182 ± 0.025	0.154 ± 0.037	0.148 ± 0.014	0.144 ± 0.020
C16:1	14.977± 0.320	14.619 ± 0.985	14.021 ± 1.436	14.297± 0.820
C17:1	0.404 ± 0.059	0.421 ± 0.052	0.418 ± 0.024	0.420 ± 0.026
C18:1	34.496 ± 1.670	32.640 ± 0.932	34.092 ± 0.602	34.794 ± 1.104
C20:1	1.326 ± 0.080	1.427 ± 0.040	1.342 ± 0.082	1.350 ± 0.053
C22:1	0.077 ± 0.015	0.077 ± 0.017	0.084 ± 0.018	0.077 ± 0.005
∑Monounsaturated fatty acids (MUFAs)	51.461 ± 1.953	49.336 ± 1.864	50.104 ± 1.012	51.083 ± 0.360
C18:2 n-6	10.820 ± 1.131	12.447 ± 1.057	12.799 ± 1.822	11.482 ± 0.895
C20:2 n-6	0.248 ± 0.002 ^a^	0.276 ± 0.023 ^b^	0.247± 0.010 ^ab^	0.246 ± 0.010 ^ab^
C20:3 n-6	0.782± 0.037	0.918 ± 0.458	0.780± 0.111	0.820 ± 0.110
C20:4 n-6	0.079 ± 0.016	0.087 ± 0.011	0.071 ± 0.005	0.078± 0.013
C18:3 n-3	1.440 ± 0.115	1.558 ± 0.115	1.404 ± 0.099	1.473 ± 0.090
C20:5 n-3	2.789 ± 0.157	3.069 ± 0.271	2.926 ± 0.410	2.989 ± 0.096
C22:6 n-3	5.015 ± 0.317 ^a^	5.666 ± 0.025 ^b^	5.325 ± 0.430 ^ab^	5.322 ± 0.160 ^ab^
∑Polyunsaturated fatty acids (PUFAs)	21.173 ± 1.443	24.021 ± 1.840	23.551 ± 2.523	22.410 ± 0.819
∑Unsaturated fatty acids (UFAs)	72.634 ± 0.534	73.358 ± 0.074	73.655 ± 1.268	73.494 ± 0.697
∑Atherosclerotic index (AI)	0.427 ± 0.011	0.405 ± 0.017	0.401 ± 0.029	0.401 ± 0.015
∑Thrombosis index (TI)	0.442 ± 0.018	0.409 ± 0.006	0.416 ± 0.046	0.416 ± 0.019

Note: Data are presented as means ± SD (*n* = 3). Different letters represent significant differences between different salinity groups at *p* < 0.05.

**Table 4:** Effect of salinity on the nucleotides in *Aplodinotus grunniens* muscle.

Nucleotides	Taste Threshold	Salinity (ppt)			
		0	4	8	12
Adenosine-5′-monophosphate (AMP) (mg/100 g)	50.000	13.705 ± 0.877	13.686 ± 1.662	13.595 ± 0.316	12.653 ± 0.353
Guanosine-5′-monophosphate (GMP) (mg/100 g)	12.000	1.674 ± 0.119 ^a^	1.986 ± 0.125 ^bc^	2.135 ± 0.202 ^c^	2.453 ± 0.134 ^cd^
Inosine-5′-monophosphate (IMP) (mg/100 g)	25.000	41.352 ± 4.608 ^a^	57.377 ± 4.257 ^b^	127.209 ± 15.193 ^c^	164.468 ± 12.721 ^d^
Taste activity value (TAV) of IMP	/	1.654 ± 0.184 ^a^	2.295 ± 0.170 ^b^	5.088 ± 0.608 ^c^	6.579 ± 0.509 ^d^
Equivalent umami concentration (EUC) (g MSG/100 g)	/	1.067 ± 0.242 ^a^	1.829 ± 0.541 ^a^	3.850 ± 0.617 ^b^	6.534 ± 0.948 ^c^

Note: Data are presented as means ± SD (*n* = 3). Different letters represent significant differences between different salinity groups at *p* < 0.05.

## Data Availability

Data are contained within the article.
